# Cigarette Smoke Extract (CSE) Delays NOD2 Expression and Affects NOD2/RIPK2 Interactions in Intestinal Epithelial Cells

**DOI:** 10.1371/journal.pone.0024715

**Published:** 2011-09-12

**Authors:** Marian C. Aldhous, Kimberley Soo, Lesley A. Stark, Agata A. Ulanicka, Jennifer E. Easterbrook, Malcolm G. Dunlop, Jack Satsangi

**Affiliations:** 1 Gastrointestinal Unit, Molecular Medicine Centre, Western General Hospital, University of Edinburgh, Edinburgh, Scotland, United Kingdom; 2 Edinburgh Cancer Centre and MRC Human Genetics Unit, Institute of Genetics and Molecular Medicine, Western General Hospital, Edinburgh, Scotland, United Kingdom; Johns Hopkins School of Medicine, United States of America

## Abstract

**Background:**

Genetic and environmental factors influence susceptibility to Crohn's disease (CD): NOD2 is the strongest individual genetic determinant and smoking the best-characterised environmental factor. Carriage of NOD2 mutations predispose to small-intestinal, stricturing CD, a phenotype also associated with smoking. We hypothesised that cigarette smoke extract (CSE) altered NOD2 expression and function in intestinal epithelial cells.

**Methods and Findings:**

Intestinal epithelial cell-lines (SW480, HT29, HCT116) were stimulated with CSE and nicotine (to mimic smoking) ±TNFα (to mimic inflammation). NOD2 expression was measured by qRT-PCR and western blotting; NOD2-RIPK2 interactions by co-immunoprecipitation (CoIP); nuclear NFκB-p65 by ELISA; NFκB activity by luciferase reporter assays and chemokines (CCL20, IL8) in culture supernatants by ELISA. In SW480 and HT29 cells the TNFα-induced NOD2 expression at 4 hours was reduced by CSE (p = 0.0226), a response that was dose-dependent (p = 0.003) and time-dependent (p = 0.0004). Similar effects of CSE on NOD2 expression were seen in cultured ileal biopsies from healthy individuals. In SW480 cells CSE reduced TNFα-induced NFκB-p65 translocation at 15 minutes post-stimulation, upstream of NOD2. Levels of the NOD2-RIPK2 complex were no different at 8 hours post-stimulation with combinations of CSE, nicotine and TNFα, but at 18 hours it was increased in cells stimulated with TNFα+CSE but decreased with TNFα alone (p = 0.0330); CSE reduced TNFα-induced NFκB activity (p = 0.0014) at the same time-point. At 24 hours, basal CCL20 and IL8 (p<0.001 for both) and TNFα-induced CCL20 (p = 0.0330) production were decreased by CSE. CSE also reduced NOD2 expression, CCL20 and IL8 production seen with MDP-stimulation of SW480 cells pre-treated with combinations of TNFα and CSE.

**Conclusions:**

CSE delayed TNFα-induced NOD2 mRNA expression and was associated with abnormal NOD2/RIPK2 interaction, reduced NFκB activity and decreased chemokine production. These effects may be involved in the pathogenesis of small-intestinal CD and may have wider implications for the effects of smoking in NOD2-mediated responses.

## Introduction

The chronic inflammatory bowel diseases (IBD), Crohn's disease (CD) and ulcerative colitis (UC) are now common causes of gastrointestinal disease in the UK, estimated at 1 in 250 [1]. The aetiology of IBD is unknown, but dysregulated innate intestinal responses to luminal bacteria are consistently implicated in animal and human studies of disease pathogenesis [2]. Recent genome-wide association studies identified a number of susceptibility genes contributing to the pathogenesis of IBD [3], [4]. These findings are consistent with the model that CD and UC are related polygenic diseases, sharing some but not all genetic determinants [5].

Nucleotide-binding oligomerization domain (NOD)2 is an intracellular receptor for the bacterial motif muramyl-dipeptide (MDP) [6]. NOD2 interacts with receptor interacting serine-threonine kinase-2 (RIPK2, also known as RIP2, RICK or CARDIAK) through the interaction of their CARD domains, leading to the poly-ubiquitination of RIPK2 and activation of the transcription factor NFκB [7]. NOD2 was the first susceptibility gene identified in CD [8], [9] and remains the strongest genetic determinant yet discovered. Disease-associated NOD2 mutations occur in CD patients, with associations with small bowel CD [10] and complications of stricture and fistula formation [11], [12] but not with colonic CD or UC. The common NOD2 mutations have also found to be important in other diseases, e.g. in bone-marrow transplant; there is some evidence that NOD2 mutations enhance the occurrence and/or severity of intestinal symptoms in graft versus host disease (GvHD) [13]; they have also been shown to be important in susceptibility to leprosy [14], [15] and colorectal cancer [16].

The mechanisms whereby NOD2 mutations result in intestinal inflammation in CD remain incompletely understood. NOD2 is expressed by professional antigen presenting cells (APC), as well as a variety of other cell types. In the intestine, these include epithelial cells, Paneth cells and goblet cells. Evidence suggests that NOD2 mutations are associated with a loss of innate immune protective mechanisms in both circulating APC and in the intestine: e.g. loss of cellular NOD2 expression [17] and downstream NFκB signalling [17], [18], reduction of the α-defensins from Paneth cells [19], cytokine production [20] and intestinal epithelial cell barrier function [21]. NOD2 has been shown to interact with the autophagy protein, ATG16L1 (another genetic susceptibility locus for CD [3]), in the response to bacteria [22], [23]. The role of NOD2 is wider than previously thought as it has also been shown to be expressed by neutrophils [24] and to have anti-viral responses [25]. The relative importance of altered NOD2 signalling in circulating APC compared with intestinal epithelial cell NOD2 signalling in the pathogenesis of CD remains a critical area for investigation.

NOD2 in intestinal epithelial cells is important in maintenance of barrier function against bacteria [26], [21]. The immune response to bacteria is initiated by macrophages and dendritic cells in the *lamina propria* sampling the gut lumen. These cells produce pro-inflammatory cytokines, including TNFα [27], [28], which increase NOD2 expression in nearby epithelial cells [29], suggesting that NOD2 up-regulation with inflammation is an important and appropriate *initial* response within these cells, to “prime” cells to increase antibacterial responses; indeed NFκB response elements within the NOD2 promoter are involved in increased NOD2 expression [18]. Intestinal epithelial cell responses to muramyl-dipeptide (MDP) have been found to be more efficient in cells pre-treated with TNFα to induce NOD2 [29]. Adherent invasive *E. coli* (AIEC) have been isolated from ileal CD. These bacteria colonise epithelial cells and are also able to induce the secretion of large amounts of TNFα from macrophages [26]. The increase in expression of NOD2 in these cells leads to the production of cytokines and anti-microbial peptides [30], also important in the anti-bacterial response.

Apart from the bacterial environment, cigarette smoking is the best known environmental factor to influence IBD aetiology: smoking is associated with increased incidence and severity of CD but prevention of development of UC [31], [32]. The mechanisms and smoke constituent(s) involved require further investigation; only nicotine has received attention in IBD, in clinical trials or laboratory studies [33], [34]. Nicotine has profound effects on immune and GI mechanisms relevant to IBD [35], [36]. The effects of nicotine or other components of cigarette smoke on gut-derived cells are poorly characterised. Nicotine levels in saliva and gastric juice of smokers have been found to be considerably higher than that found in blood [35], suggesting that cigarette smoke products dissolve quickly in saliva and are swallowed. Cigarette smoke extract (CSE) has been used as a model for the effects of smoking in lung diseases and shown to increase production of pro-inflammatory cytokines [37], affect apoptosis [38], neutrophil phagocytosis [39] and mechanisms of lung repair [40], [41], all of which are relevant to the gut.

We have previously shown profound effects of smoking on disease history in both CD and UC, whereby smoking defined the disease location or extent and subsequent disease course [42], [43]. Cessation of smoking induces a more benign disease course in CD patients [44]. While there is no direct association between NOD2 variants and smoking habit [45], CD patients who smoke tend to have a phenotype similar to those with NOD2 mutations: stricturing, ileal disease [10], [11], [12], raising the question of whether smoking might affect NOD2 expression or activation in intestinal cells.

We hypothesised that constituents of cigarette smoke may have a direct effect on innate immune activation in the intestinal epithelium, by affecting NOD2 signalling. The primary aim of this study was to investigate whether CSE or nicotine affected NOD2 expression in intestinal epithelial cell lines (SW480, HT29 and HCT116) and biopsies from healthy individuals. SW480 and HCT116 cells are known to constitutively express NOD2 [21]; NOD2 is induced in HT29 and up-regulated in SW480 cells by TNFα, which we used to model the inflammation-induced up-regulation of NOD2 [29]. We demonstrate that cigarette smoke extract (CSE) was responsible for a marked inhibition of TNFα-induced NOD2 expression, with a reduction in the early translocation of NFκB. We also show that CSE prolonged the NOD2-RIPK2 interaction with a concomitant reduction in NFκB activity. CSE also inhibited chemokine production from these cells. Treatment of cells with combinations of TNFα, nicotine and CSE prior to stimulation with MDP also affected NOD2 expression and chemokine production. All these responses potentially give new insight into the mechanisms behind cigarette smoking and CD and may have wider implications for the effect of smoking on other NOD2-mediated immune mechanisms.

## Methods

Unless otherwise specified all reagents were obtained from Invitrogen, UK.

### Cigarette smoke extract (CSE) and cell stimulation

Intestinal epithelial adenocarcinoma cell lines were obtained from the European Collection of Cell Cultures (HPACC, UK) and grown in media containing Penicillin (100 U/ml), Streptomycin (0.1 mg/ml), Glutamine (1 mM) and 10% Foetal Bovine serum. SW480 cells were grown in Leibovitz L15 medium, HCT116 cells in McCoy's medium and HT29 cells in DMEM.

CSE was freshly made based on a method for cigarette smoke condensate [46]. Smoke from one cigarette (Regal King-size, containing 10 mg Tar and 0.9 mg nicotine) was bubbled through sterile PBS (2.5 ml), and the resulting solution (denoted 100% CSE) used to stimulate cells at a final concentration of 2%, similar to amounts used in studies of CSE on signalling pathways [46], [47]. Cells were stimulated with TNFα (50 ng/ml, Peprotech, UK) and nicotine at 100 ng/ml (Sigma, UK) which approximated to that found in arterial blood [35] and in cervical mucus [48], another mucosal site distant from the lung. Unless otherwise stated, cells were stimulated for 4 hours, harvested and stored at −80°C prior to RNA extraction. Experiments were also carried out for 24 hours and supernatants were stored at −80°C. For some experiments, cells were pre-treated with combinations of TNFα, CSE and nicotine for 4 hours and the medium changed prior to stimulation with MDP (L18-MDP, 1 µg/ml, Invivogen, UK) for a further 4 or 24 hours.

Cells were stimulated with other chemicals from cigarette smoke at concentrations previously found to affect NFκB activation: acrolein (10 µM, Sigma, UK) [49], 4-hydroxy nonenal (HNE, 10 µM, Alexis Chemicals, UK) [50], or hydrogen peroxide (HP, 100 µM, Sigma, UK) [46]. For CSE titration experiments, SW480 cells were stimulated with TNFα alone or TNFα with CSE diluted to 2%, 1%, 0.5%, 0.25%, 0.125% and 0.0625%. For the time-courses SW480 cells were stimulated with TNFα ±2% CSE for 0.5 and 1 hour and at hourly intervals up to 8 hours.

### Ethics Statement

Ethical approval for this study was obtained from Lothian Ethics Committee (LREC 2001/4/72, amended July 2009). All individuals gave informed consent.

### Organ culture of intestinal biopsies

Ileal biopsies were obtained from otherwise healthy individuals (n = 10) undergoing routine endoscopy for cancer surveillance due to family history or previous polyps. The mean age of the individuals at sampling was 48.3 years (range 23–71 years). Smoking status was current smoker (n = 2), never-smoker (n = 5), ex-smoker (n = 3). One biopsy was stored as an uncultured control; four other biopsies were cultured *ex vivo* in an organ culture model, as previously described [34] and stimulated with CSE (0.5%), TNFα (50 ng/ml), CSE+TNFα or medium only, for 24 hours. Biopsies were harvested and stored in RNA Later® at −80°C prior to RNA extraction.

### Quantitative RT-PCR for NOD2 and RIPK2

RNA was extracted from cells using RNeasy® kits (Qiagen, UK). RNA (1 µg) was transcribed to cDNA using the SuperScriptIII® Reverse Transcriptase cDNA kit with a 1∶1 mixture of oligo-dT_20_ and random hexamers as primers.

Initial PCRs for NOD2, GAPDH (housekeeping gene) and all RIPK2 PCRs were carried out on the Rotorgene®6000 (Corbett Research Instruments [now Qiagen], UK). PCR mix was: EXPRESS SYBR® GreenER qPCR Supermix with premixed ROX containing 1 µM of each primer (Sigma Genosys, UK) and template cDNA, with RNA-negative and water controls. Primer pairs were chosen using Primer3 and crossed exons. Primer sequences: NOD2: forward AAGCAAGAGTCTGGTGTCCCTG, reverse GGGGCAACAGAGTGGGTGAC; RIPK2: forward GGGATAGCACCATTTCTGGA, reverse TGGCAAATTCTTCTCCTTGG; GAPDH: forward TCATCTCTGCCCCCTCTGCT; reverse CGACGCCTGCTTCACCACCT; qPCR cycle: 50°C for 2 minutes, 95°C for 2 minutes and 40 cycles of 95°C for 15 s, 60°C for 1 minute. Initial gain optimisation and final melt-curve analysis (60°–95°C) were included. Subsequent PCR experiments for NOD2 and GAPDH used Taqman® expression assays (Hs00223394_m1, Hs99999905_m1, respectively) according to manufacturer's protocol. cDNA from SW480 cells was used as a standard curve, with the same DNA dilutions for NOD2, RIPK2 and GAPDH. PCR quantification used ΔΔct method for the gene of interest (NOD2 or RIPK2) against the normaliser gene (GAPDH). For each cell line, unstimulated cells were used as the calibrator (assigned a value of 1); other samples' gene expression values were calculated relative to the expression in unstimulated cells.

### NFκB-p65 ELISA

SW480 cells were stimulated for 15 minutes with TNFα ± CSE or nicotine and harvested on ice. Nuclear extracts were made using a Nuclear Extract Kit (Active Motif, Belgium). The nuclear proteins were analysed in duplicate on an NFκB-p65 ELISA kit (Active Motif, Belgium).

### Co-immunoprecipitation for NOD2-RIPK2 interaction

Full-length wild-type NOD2 cDNA was cloned into a pCMV-myc vector (Clontech, UK) and sequenced to check expression. SW480 cells were transfected with plasmid DNA (5 µg), using Lipofectamine 2000™. After overnight recovery, cells were stimulated with combinations of CSE, nicotine and TNFα for 8 and 18 hours. Cells were harvested and lysed on ice for 20 minutes in NP-40 Lysis buffer (500 µl, containing: 0.1 M NaCl [Sigma, UK], 0.5% NP40-alternative [Calbiochem, UK], 0.5 M Hepes pH 7.4, 0.01 M EDTA [both from Sigma, UK] and Complete™ protease-inhibitors [Roche, UK]). After centrifugation (18000×*g* for 15 minutes at 4°C), protein concentrations were measured and equalised between samples. Samples were mixed overnight with RIPK2 antibody (2 µg, Abgent, UK) and Protein G agarose beads (Roche, UK) on an orbital mixer at 4°C. Beads (and immunocomplex) were collected by centrifugation and washed 3 times in PBS. Sample buffer (20 µl, 50% 4× NuPAGE LDS loading buffer, 40% PBS and 10% 2-mercaptoethanol [Sigma, UK]) was added to the beads and boiled for 5 minutes to dissociate immunocomplexes, which were collected by centrifugation.

### Western blotting

The immunocomplexes and initial cell lysates from the CoIP experiments were run on NuPAGE® Novex® 4–12% Bis-Tris gels in MOPS SDS running buffer at 200 V for 50 minutes. Proteins were transferred onto PVDF membrane using NuPAGE® Transfer buffer at 30 V for 1 hour. Blots were blocked overnight in 5% milk (Marvel, Cadbury's, UK) in PBS/0.1% Tween20 at 4°C. Primary antibodies diluted in 5% milk/PBS/0.1% Tween20 were used to probe for myc (1∶100, sc40, Santa Cruz, USA), RIPK2 (1∶100, rabbit anti-human RIPK2 antibody, Abgent, UK) and β-Actin (1∶100, sc-69879, Santa-Cruz, USA) and incubated overnight at 4°C. Secondary antibodies were used at 1∶1000 for 2 hours at room temperature. Bands were visualised using ECL reagent on photographic film. NOD2-myc, RIPK2 and β-actin bands were quantified by densitometry. For the western blots and CoIPs the NOD2∶β-actin or NOD2∶RIPK2 ratios, respectively, were reported relative to unstimulated cells (assigned a value of 1).

### Reporter Assays

The plasmids used have been described elsewhere [51]. The NFκB construct had three NFκB binding sites, which were deleted in the ΔκB construct. Transfection efficiency was measured using a pCMV-β-galactosidase construct (PCMV-β, Promega, UK). Cells were co-transfected with the pCMV-β plasmid and either the NFκB or ΔκB plasmids using Lipofectamine™ 2000. After overnight recovery cells were stimulated with combinations of CSE, nicotine (10 µg/ml) and TNFα, or with acrolein, HNE, or HP ± TNFα. After 18 hours, cells were harvested and lysed using a reporter lysis assay kit (Promega, UK). Luciferase activity was measured on a LB9507 Luminometer (Berthold). β-galactosidase was measured using a β-galactosidase activity kit (Promega, UK). NFκB activity was calculated as units of luciferase activity per unit of β-galactosidase activity and reported relative to unstimulated cells (assigned a value of 1).

### ELISAs for CCL20 and IL8

CCL20 and IL8 were measured in culture supernatants of the 24-hour samples using matched antibody pairs (DuoSets, R&D Systems, UK).

### Statistics

Comparisons of results of different stimuli were compared by a non-parametric, one-way ANOVA for repeated measures (Friedman test) or Kruskall-Wallis for biopsies. Comparison of results across cell lines or time points used a two-way ANOVA. All statistical tests were carried out using GraphPad Prism4® GraphPad Software, San Diego, CA).

## Results

Results from cells stimulated with CSE alone are denoted ***C***, nicotine alone as ***N***, CSE and nicotine together as ***CN***, TNFα alone as ***T***, TNFα and CSE together as ***TC***, TNFα and nicotine together as ***TN***, TNFα, CSE and nicotine together as ***TCN***.

### CSE inhibited TNFα-induced up-regulation of NOD2 expression at 4 hours

**Figure 1:**
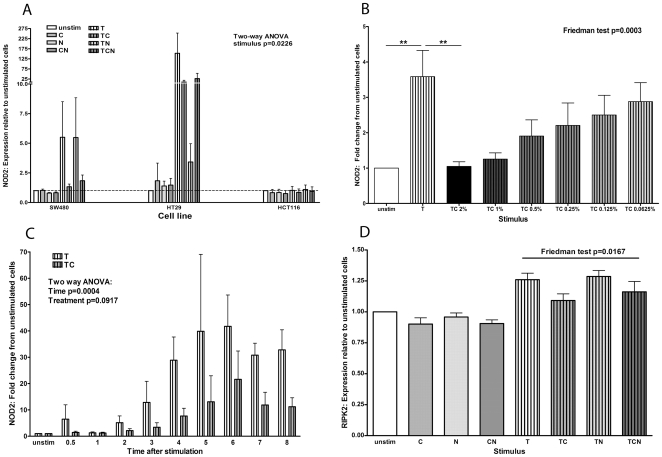
TNFα-induced NOD2 and RIPK2 expression is reduced by CSE. **A**: Epithelial cell lines (SW480, HT29, HCT116) were stimulated with combinations of 2% CSE (***C***), 100 ng/ml nicotine (***N***) and 50 ng/ml TNFα (***T***). NOD2 mRNA expression is shown in response to these stimuli as relative expression compared with unstimulated (unstim) cells, which were given a value of 1. Results from cells stimulated with CSE alone are denoted ***C***, nicotine alone as ***N***, CSE and nicotine together as ***CN***, TNFα alone as ***T***, TNFα and CSE together as ***TC***, TNFα and nicotine together as ***TN***, TNFα, CSE and nicotine together as ***TCN***. **B**: The reduction in TNFα-induced NOD2 expression is dose-dependent. SW480 cells were stimulated with ***T*** or ***TC*** at decreasing percentage solution concentrations from 2% (TC 2%) down to 0.0625% (TC 0.0625%). NOD2 mRNA expression is shown as detailed in A. Bars denote significant differences (*post-hoc* Dunn's test ***T*** vs unstim and ***T*** vs ***TC*** 2%, p<0.01). **C**: Time course of TNFα-induced NOD2 expression. SW480 cells were stimulated with ***T*** or ***TC*** for time intervals up to 8 hours. The NOD2 expression at different time-points is shown as detailed in A. **D**: RIPK2 expression in SW480 cells is shown as detailed in A.

TNFα up-regulated NOD2 expression in SW480 (mean relative expression [RE] = 5.5) and HT29 cells (mean RE = 153); ***C***, ***N*** or ***CN*** had little effect on NOD2 expression in any cell line (fig. 1A). Compared with ***T***, NOD2 expression was reduced by ***TC*** (mean RE from 5.5 to 1.3) in SW480 and HT29 cells (mean RE 115 to 10.3); ***TN*** had no effect compared with ***T*** in SW480 cells (mean RE 5.5 to 5.5) but reduced expression in HT29 cells (mean RE 115 to 3.4). ***TCN*** reduced NOD2 expression in both SW480 (mean RE 5.5 to 1.8) and HT29 cells (mean RE 115 to 27.0, figure 1A, two-way ANOVA for stimulus p = 0.0226). There was little change in NOD2 expression in HCT116 cells at 4 hours regardless of the stimulus used. Because of this and the very low constitutive NOD2 expression in HT29 cells (increasing the level of error due to small changes in the unstimulated samples), we used the SW480 cell line for subsequent experiments.

In SW480 cells, the reduction in TNFα-induced NOD2 expression by CSE was dose-dependent: titration of CSE from 2% to 0.0625% showed the restoration of the TNFα-induced NOD2-response as the amount of CSE decreased (figure 1B, Friedman test p = 0.0003). Time course experiments comparing ***T*** with ***TC*** showed that ***T***-induced NOD2 expression was evident at 2–3 hours and peaked at 5–6 hours (figure 1C), whereas treatment with ***TC*** at the same time points showed that the expression of NOD2 was reduced and significantly delayed (two-way ANOVA for time p = 0.0004).

NOD2 interacts with RIPK2 to initiate NFκB activation [7]. qPCR results for RIPK2 (figure 1D, from 5 experiments) showed that RIPK2 expression was not increased by ***C***, ***N*** or ***CN***. ***T*** and ***TN*** marginally increased RIPK2 expression (mean RE was 1.3 for both), which was not seen with ***TC*** or ***TCN*** (Friedman test p = 0.0167, post hoc test ***TC*** vs ***TN*** p<0.05).

### CSE reduced translocation of NFκB p65 at 15 minutes

**Figure 2:**
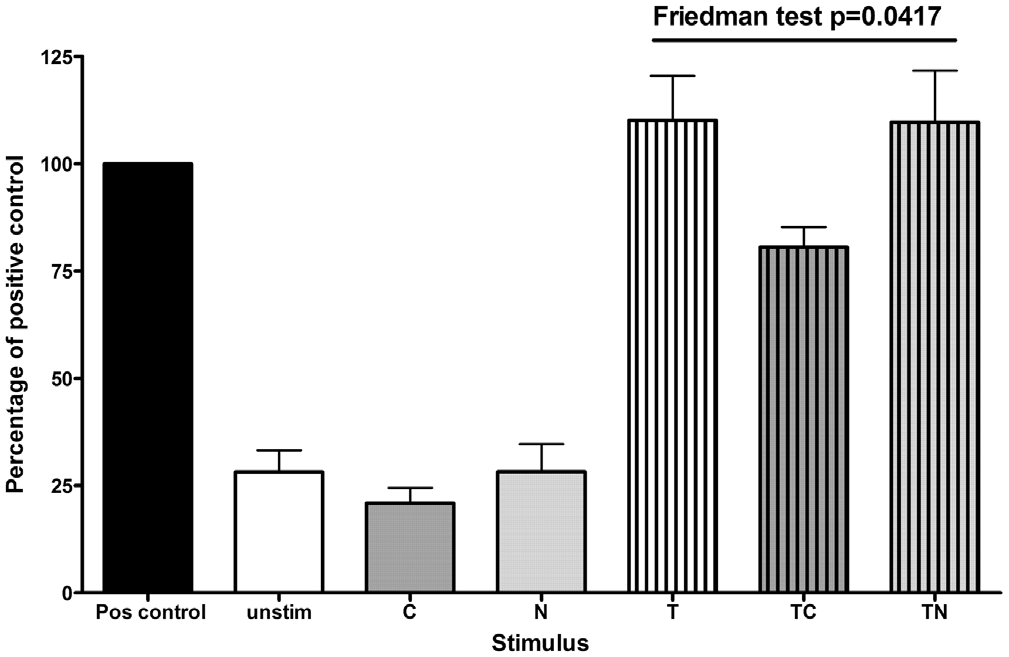
TNFα-induced translocation of NF-κB-p65 is reduced by CSE. Nuclear NF-κB-p65 levels were measured by ELISA in cells stimulated with CSE (***C***), nicotine (***N***) ± TNFα (***T***), as detailed in figure 1A. Results of 4 experiments are shown as percentage of the positive control (pos control). A Friedman test showed a significant decrease in NF-κB-p65 in cells stimulated with ***TC*** compared with ***T*** and ***TN***.

The NOD2 promoter contains two functional NFκB binding sites which are involved in the TNFα-induced NOD2 expression [18]. To investigate whether CSE or nicotine affected TNFα-induced NFκB-p65 translocation from cytoplasm to nucleus and thus potentially affect NOD2 expression, we used an NFκB-p65 ELISA on nuclear extracts of cells stimulated for 15 minutes. Results are expressed as percentage of the positive control (100%, figure 2, from 4 experiments). Unstimulated, ***C***- or ***N***-treated cells had low levels of NFκB-p65 (∼25% of positive control); ***T***- and ***TN***- treated cells had high levels of NFκB-p65 (110% of positive control), whereas NFκB-p65 in ***TC***-treated cells (80% of positive control) were significantly lower than those of ***T*** and ***TN*** (Friedman test, p = 0.0417).

### CSE increased TNFα-induced NOD2-RIPK2 co-IP complex at 18 hours

**Figure 3:**
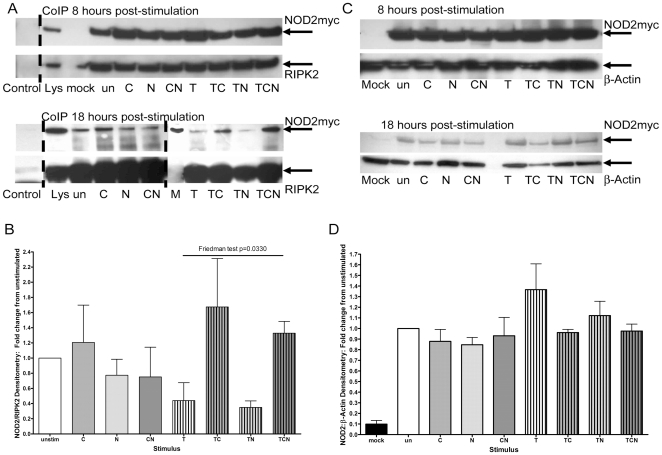
Co-immunoprecipitation and Western blot. **A & B**: *Co-immuprecipitation.* Cells were transfected with NOD2-myc and stimulated for 8 or 18 hours prior to co-IP for NOD2 and RIPK2. Representative western blots probed for NOD2-myc and RIPK2 after co-IP are shown (A). Control denotes a co-IP using an irrelevant antibody, mock denotes sham-transfected cells and Lys denotes NOD2-myc and RIPK2 levels in lysed cells prior to co-IP. Densitometry of NOD2 normalised against RIPK2 is shown from 3 experiments stimulated for 18 hours and expressed relative to unstimulated cells which were given a value of 1 (B). A Friedman test of the NOD2:RIPK2 levels showed a significant difference in those cells stimulated with ***T***, ***TC***, ***TN*** and ***TCN***. **C & D**: *Western blot.* The lysates used for the co-IPs after stimulation for 8 or 18 hours were run on western blots showed similar patterns of response to combinations of CSE, nicotine and TNFα. Representative western blot of NOD2 and β-Actin are shown for 8 and 18 hours (C). For the 18 hour experiments, densitometry normalised against β-Actin from 4 experiments (D) and is expressed as detailed in B.

We wished to investigate whether CSE affected NOD2-RIPK2 protein interactions. Due to the unavailability of a reliable anti-NOD2 antibody we used cells transfected with a NOD2-myc construct and an anti-myc antibody. We carried out co-IP experiments after NOD2-transfection and stimulation for 8 and 18 hours. At 8 hours there was no significant difference in the amount of NOD2-myc/RIPK2 complex in the cells treated with different stimuli, whereas after 18 hours cells treated with ***T*** and ***TN*** had smaller amounts of NOD2-myc/RIPK2 complex compared with cells treated with ***TC*** and ***TCN*** (figure 3A, representative blots of CoIPs for 8 and 18 hours; fig. 3B for densitometry against RIPK2 for 18 hours, from 3 experiments). For unstimulated cells and those stimulated with ***C***, ***N*** and ***CN*** there was little change in the amount of NOD2-myc/RIPK2 complex. At 18 hours, the relative amounts of NOD2-myc/RIPK2 in cells stimulated with ***T***, ***TC***, ***TN*** and ***TCN*** were significantly different (Friedman test, p = 0.0330).

To determine whether differences in CoIP reflected differences in protein expression, western blots were run from the same lysates. At 8 hours after stimulation with combinations of TNFα, CSE and nicotine, NOD2-myc protein expression was no different. After 18 hours, although changes were not significant, a similar pattern to that seen with mRNA was found: increased NOD2-myc protein with ***T***- and ***TN***-stimulation but reduced to background levels with ***TC***, ***TCN***, and no effect of ***C***, ***N*** or ***CN*** (figure 3C, representative blots for 8 and 18 hours; fig. 3D densitometry against β-actin for 18 hours from 3 experiments).

### CSE reduced TNFα-induced NFκB activity

**Figure 4:**
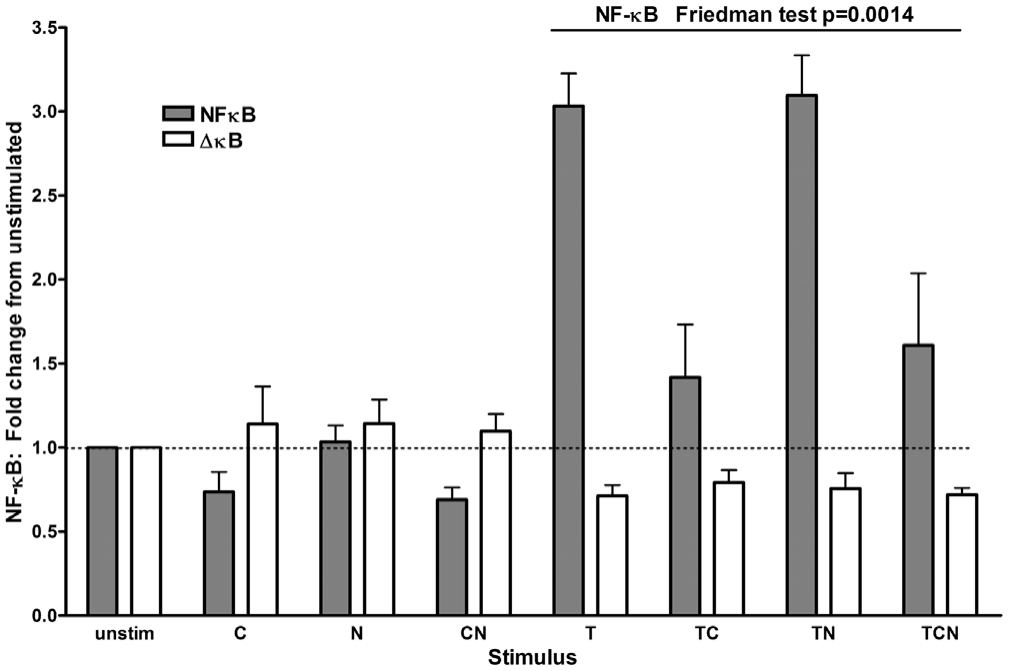
TNFα-induced NFκB activity at 18 hours is significantly reduced by CSE. Cells transfected with reporter constructs for NFκB (grey bars) or those with the NFκB binding sites removed (ΔκB, white bars) were stimulated for 18 hours with combinations of CSE (***C***), nicotine (***N***) and TNFα (***T***). Results (of 5 experiments) were normalised against a transfection control and NFκB activity is expressed relative to that in unstimulated cells, which were given a value of 1. A Friedman test showed a significant different in those cells stimulated with ***T***, ***TC***, ***TN*** and ***TCN***.

Reporter assays were used to assess whether CSE, TNFα and nicotine affected NFκB activation at 18 hours. ***C***, ***N*** and ***CN*** had little effect on NFκB activity. ***TC*** and ***TCN***, but not ***TN***, significantly reduced ***T***-induced NFκB activity (Friedman test p = 0.0014, figure 4).

### CSE reduced basal and TNFα-induced CCL20 and basal IL8 production

**Figure 5:**
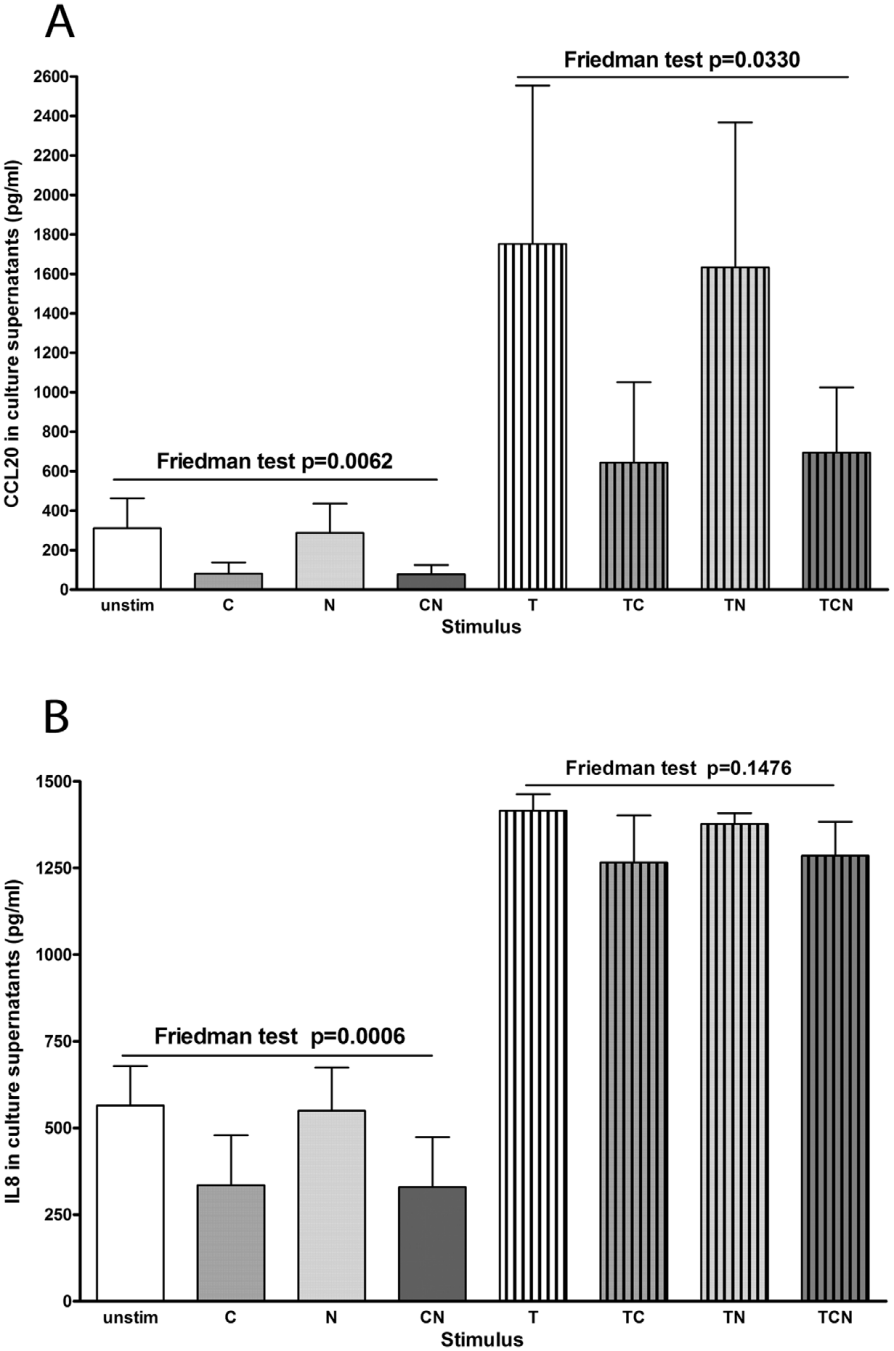
CSE reduced basal and TNFα-induced CCL20 and IL8 production at 24 hours. Supernatants from cells stimulated with combinations of CSE (***C***), nicotine (***N***) and TNFα (***T***) were harvested after 24 hours and CCL20 and IL8 were measured by ELISA. Results of 3 experiments are shown. Friedman tests showed significant differences in CCL20 and IL8 production in cells stimulated with ***C***, ***N*** and ***CN*** against unstimulated cells. A significant difference in CCL20 production was seen in cells stimulated with ***T***, ***TC***, ***TN*** and ***TCN***. A similar pattern was shown in for IL8 production with ***T***, ***TC***, ***TN*** and ***TCN*** but did not quite reach significance.

To determine whether combinations of CSE, TNFα or nicotine affected chemokine production, CCL20 and IL8 were assayed in supernatants of cells cultured for 24 hours after stimulation. Both CCL20 and IL8 production were significantly reduced after stimulation with ***C*** or ***CN***, but not ***N*** (figures 5A and B respectively, Friedman tests p = 0.0062 and p = 0.0006, respectively). ***T*** increased CCL20 production, which was significantly reduced by ***TC*** and ***TCN***, but not by ***TN*** (figure 5A, Friedman test p = 0.0330). ***T*** increased IL8 production, which was slightly but not significantly reduced with ***TC*** but not ***TN*** (figure 5B, Friedman test p = 0.1476). There were no significant differences in cell death with any stimulation.

### CSE reduced basal and TNFα-induced responsiveness to MDP

**Figure 6:**
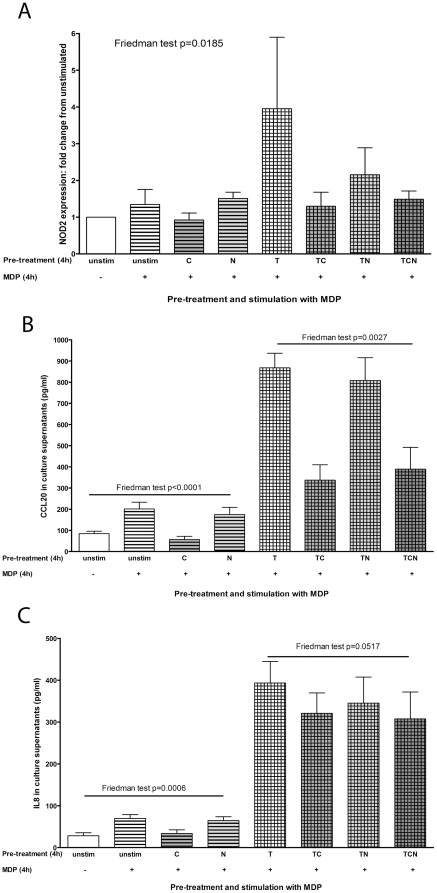
Pre-treatment with CSE and TNFα affected responses to MDP. **A**: SW480 cells were pre-treated with combinations of ***C***, ***N*** and ***T*** for 4 hours before the medium was changed and cells were stimulated with MDP (1 µg/ml, denoted by **+**) for 4 hours. NOD2 mRNA expression is shown in response to these stimuli as relative expression compared with unstimulated (unstim) cells, which were given a value of 1. A Freidman test showed significantly higher NOD2 expression was seen in cells pre-treated with **T**. **B** and **C**: Supernatants from cells pre-treated with combinations of CSE (***C***), nicotine (***N***) and TNFα (***T***) for 4 hours, prior to medium change and stimulation with MDP, were harvested after 24 hours; CCL20 (**B**) and IL8 (**C**) were measured by ELISA. Friedman tests showed significant differences in CCL20 and IL8 production in cells pre-treated with ***C*** and ***N*** against unstimulated cells, before MDP stimulation. A significant difference in CCL20 production was seen in cells pre-treated with ***T***, ***TC***, ***TN*** and ***TCN*** before MDP-stimulation. A similar pattern was shown in for IL8 production with pre-treatment with ***T***, ***TC***, ***TN*** and ***TCN*** but did not quite reach significance.

To determine whether pre-incubation with TNFα and/or CSE affected NOD2 expression induced by MDP, cells were pre-treated for 4 hours with combinations of TNFα and CSE. The medium was changed and cells were then stimulated with MDP. Pre-treatment with ***T*** significantly increased expression of NOD2 in response to MDP after 4 hours (Friedman test p = 0.0185, figure 6A), which was not seen with ***TC***, ***TN*** or ***TCN***. In parallel cultures, CCL20 and IL8 production showed similar effects: the increased chemokine production seen from stimulation with MDP alone was significantly reduced by pre-treatment with ***C*** but not ***N*** (CCL20: Friedman test p<0.0001, figure 6B; IL8: Friedman test p = 0.0006, figure 6C). Pre-treatment with ***T*** increased CCL20 production in response to MDP, which was significantly reduced by ***TC*** and ***TCN*** but not ***TN*** (Friedman test p = 0.0027, figure 6B). Pre-treatment with ***T*** increased IL8 production in response to MDP, which was reduced by ***TC*** and ***TCN*** but did not quite reach significance (Friedman test p = 0.0517, figure 6B).

### Effect of other components of cigarette smoke

**Figure 7:**
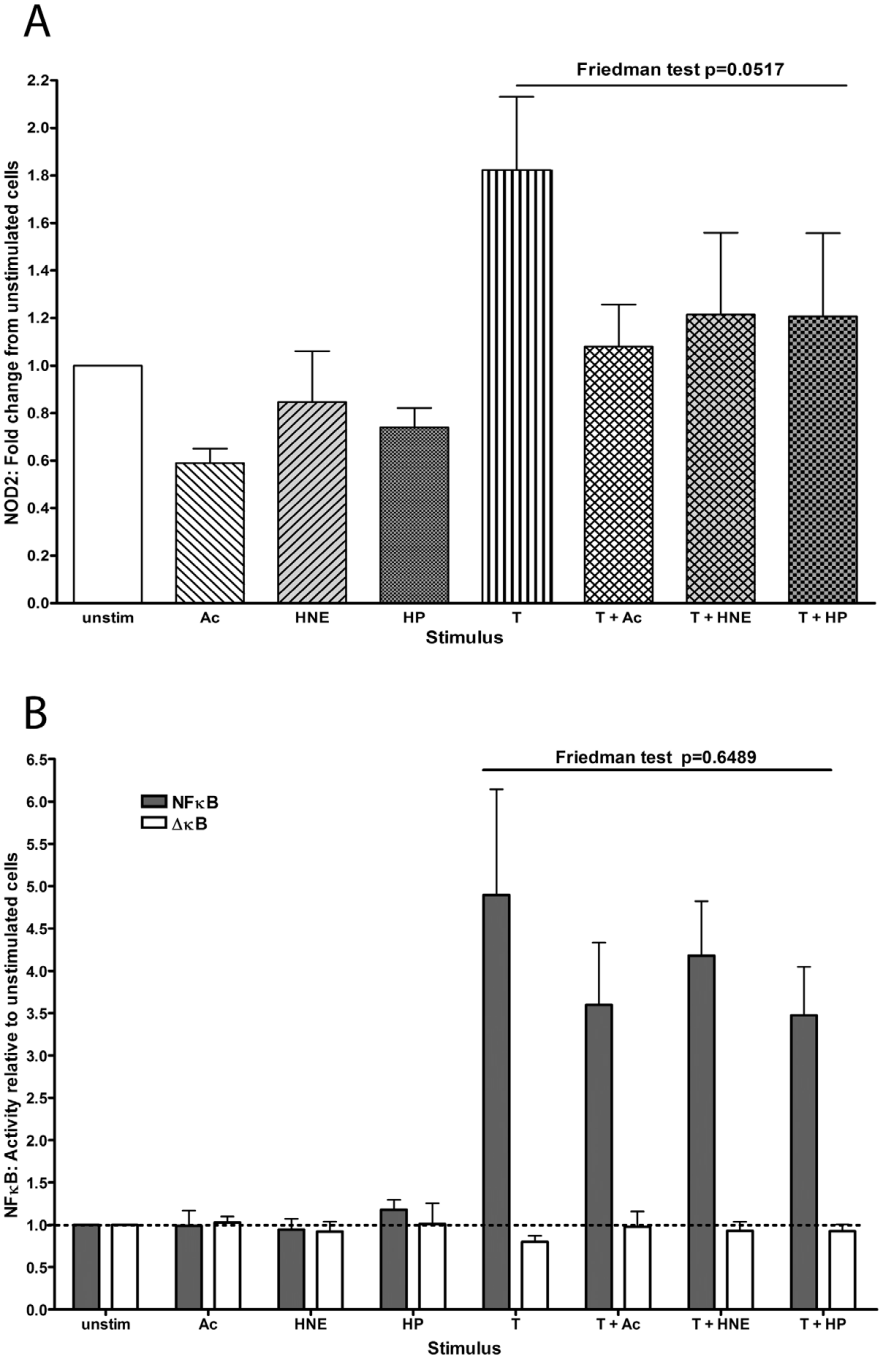
Other components of cigarette smoke had no significant effect on TNFα-induced NOD2 expression and NF-κB activity. **A**: NOD2 mRNA expression at 4 hours and **B**: NF-κB activity at 18 hours is shown for cells stimulated with other components of cigarette smoke with or without TNFα (***T***). Ac denotes Acrolein, HNE denotes hydroxyl nonenal, HP denotes hydrogen peroxide. Results are expressed relative to that in unstimulated cells, which were given a value of 1. Friedman tests showed that any differences did not reach statistical significance.

In separate experiments, cells stimulated with acrolein, HNE or HP alone did not significantly induce or decrease NOD2 expression at 4 hours, but did reduce TNFα-induced NOD2 expression (Friedman test p = 0.0517, figure 7A). Acrolein, HNE or HP alone did not affect NFκB activity. Each of them reduced TNFα-induced NFκB, but not significantly (Friedman test p = 0.6489, figure 7B).

### CSE reduced TNFα-induced expression of NOD2 in ileal biopsies from healthy controls

**Figure 8:**
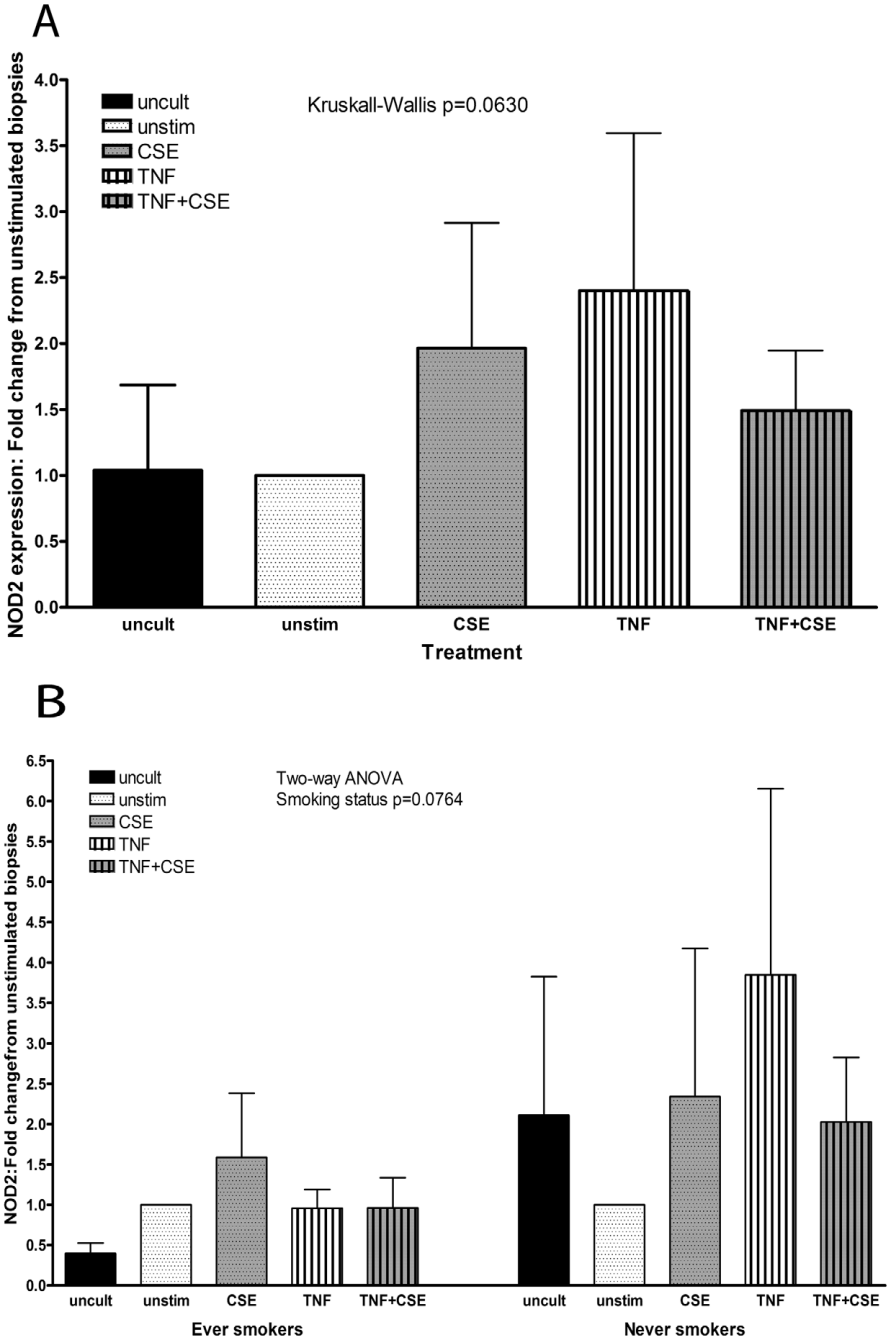
TNFα-induced NOD2 expression is reduced by CSE in cultured ileal biopsies. **A**: Biopsies from otherwise healthy individuals undergoing cancer-screening endoscopy were stimulated with combinations of CSE (0.5%) and TNFα (50 ng/ml). NOD2 mRNA expression in response to these stimuli is shown as relative expression compared with unstimulated cells, which were given a value of 1. Results from an uncultured, unstimulated biopsy per patient are also included (uncult). A Friedman test showed differences which did not quite reach statistical significance. **B**: The results from the same biopsies were compared separated according to smoking status (ever-smokers vs. non-smokers). Two-way ANOVA showed a difference according to smoking status but did not quite reach statistical significance.

To determine whether a similar pattern of the effects of TNFα and CSE on NOD2 expression was seen in ileal biopsies we cultured biopsies obtained from otherwise healthy individuals undergoing cancer-screening endoscopy (n = 10) with CSE±TNFα. In these, both CSE and TNFα increased NOD2 expression to a similar extent in ileal biopsies (mean RE = 2.0 and 2.4, respectively, figure 8A). CSE+TNFα had a lesser effect (mean RE = 1.5). None of these effects quite reached statistical significance (Kruskall-Wallis test p = 0.0630). On further analysis by smoking status, a trend was found that ever-smokers (ex- and current smokers, n = 5, figure 8B) had lower NOD2 expression than those who had never smoked (n = 5, two-way ANOVA for smoking status p = 0.0764).

## Discussion

The aims of this study were to investigate the effects of CSE and individual constituents of cigarette smoke on specific elements of the NOD2 pathway in intestinal epithelial cells. We show potentially important effects of CSE on NOD2 expression, NOD2-RIPK2 interactions, NFκB activity and chemokine production, as well as the effects on responses to MDP, all of which are pertinent to CD pathogenesis and may have wider implications for gut epithelial cell function. Whilst we cannot show whether CSE acts at only one stage or at inter-related levels, we provide compelling data to direct further studies.

NOD2 expression is known to be up-regulated in inflamed intestinal tissue of CD patients [29] and in response to TNFα *in vitro* [6], [18], [52]. We confirmed that NOD2 was constitutively expressed in SW480 and HCT116 cells and inducible in HT29 cells [21], [18]. In SW480 cells, we investigated the combined effects of pre-existing inflammation (TNFα) and/or cigarette smoke on the NOD2 response to MDP; while the time-point of 4 hours was too short to see a significant effect of MDP alone, the increased expression of NOD2 in cells pre-treated with TNFα within that time-frame confirmed that TNFα increased the NOD2 expression in response to MDP [29], a response that was negated by CSE. This suggests that CSE may reduce appropriate inflammation-induced responses to bacteria. Without MDP, CSE delayed the TNFα-induced expression of NOD2 by 2–3 hours, consistent with a decreased early TNFα-induced NFκB-p65 translocation by CSE. A study in lung epithelial cells showed that CSE decreased NFκB activation in response to *H.influenzae* [53]. Loss of NFκB activation and NFκB-dependent gene responses against pathogenic bacteria in the gut might be detrimental. Smoking may also interfere with other mechanisms that also activate NFκB in the presence of bacteria, possibly by mechanisms of oxidative stress [54] or through toll-like receptors [55].

There is a wealth of evidence that smoking habit has a profound effect on the aetiology and disease course of both CD [32], [56] and UC [42], [57]. However, the mechanisms of these effects are unclear and there is some debate as to how smoking affects the intestine. While it may at first appear counter-intuitive for inhaled cigarette smoke to affect the gut, it is highly plausible that smoke products dissolve in swallowed saliva and thus directly affect the gut, or that these products dissolve in blood and are carried to the gut through the circulation where they can exert their effects. Certainly, differences in potential mechanisms have been observed between smokers and non-smokers: differential gene expression between smokers and non-smokers was demonstrated in the descending colon of CD patients [58]. Similarly, differential DNA methylation patterns in the rectal mucosa were seen between smokers and non-smokers, which correlated with the presence of adenocarcinomas [59]. These studies strongly suggest that smoking might have a direct effect on the intestinal mucosa.

There is evidence that NOD2 is important in the maintenance of intestinal epithelial barrier function [26], [21]. NOD2 is also important in the induction of epithelial anti-microbial peptides [30]. In bone-marrow transplant, NOD2 has inhibitory effects on antigen presentation cells leading to the induction of tolerance, particularly in the gut [13]. Thus reduced NOD2 function (due to germline NOD2 mutations) has led to loss of epithelial barrier function, anti-microbial peptide production and GvHD [13], [21], [26], [30]. Similarly, reduced NOD2 expression due to smoking may also lead to loss of NOD2 function, resulting in the similar phenotypes in CD from NOD2 mutations and smoking. Our data from *ex vivo* cultured ileal biopsies of otherwise healthy individuals undergoing endoscopy for cancer surveillance suggested that TNFα-induced NOD2 expression was also reduced by CSE. The CSE effect on NOD2 expression in response to TNFα in ileal biopsies had a similar pattern to that seen with the intestinal epithelial cell lines. Interestingly, and in contrast to the cell lines, there was a NOD2 response from stimulation with CSE in the ileal biopsies. When these biopsies were separated by smoking status (ever smokers vs. never smokers), lower NOD2 expression was seen in biopsies from those patients who had ever smoked or still were smoking, raising the possibility that smoking may have a long-term down-regulatory effect on NOD2 expression.

Epithelial cells act as ‘unprofessional’ antigen presenting cells, and as such, are involved in maintaining homeostasis in the gut, leading to production of tolerogenic signals such as IL10 [60]. There is considerable interest in the role of AIEC in the pathogenesis of CD. Epithelial cells that express mutant NOD2 are less able to prevent colonisation by AIEC [26], [21]. A recent study in NOD2-deficient mice showed that *Helicobacter hepaticus* infection induced granulomatous inflammation of the ileum, which was prevented by restoration of α-defensin production [61]. Thus NOD2 mutations (and by implication decreased NOD2 expression/function through smoking) may lead to chronic inflammation uncontrolled by tolerogenic or anti-inflammatory signals [62]. It would be interesting and pertinent to see if CSE has a similar response in ‘professional’ APCs, such as circulating monocytes and tissue-derived macrophages and dendritic cells. A recent study showed that NOD2 mutations also led to the loss of IL10 production from macrophages and subsequent loss of tolerogenic mechanisms [63]. A recent review of the effects of tobacco smoke on macrophages (from lung, blood or cell lines) found conflicting results from a large number of studies, due to differences in preparation of the smoke products, the source of macrophages and the bioassays used. However, in three studies of blood-derived macrophages, smoke products induced a pro-inflammatory response, and in nine studies of lung macrophages, smoke inhibited or delayed the pro-inflammatory response of macrophages to LPS or endotoxin mediated by TLR4 [55].

Surprisingly, the NOD2-RIPK2 co-IPs showed a reduced amount of NOD2-RIPK2 complex at 18 hours from TNFα-stimulated cells, while at 8 hours the amounts of NOD2-RIPK2 complex was little different; there was also a 3-fold induction of downstream NFκB activity in cells stimulated with TNFα for 18 hours. Taken together, this suggests that the NOD2-RIPK2 complex had activated NFκB and dissociated. In contrast, cells treated with TNFα+CSE had increased amounts of the NOD2-RIPK2 complex and reduced NFκB activity, suggesting that the NOD2-RIPK2 complex had not fully activated NFκB; this is also consistent with the delayed NOD2 expression at mRNA level. The western blot results showed that it was not due to differences in the expression of NOD2-myc, but was possibly due to some interference with the NOD2-RIPK2 interactions. The NOD2-RIPK2 interaction leads to the polyubiquitination of NEMO, a key component of the IKK complex with subsequent activation of NFκB [64], [7]. The mechanisms by which CSE or smoking might interfere with these interactions, thus affecting NOD2-induced NFκB signalling, require further in depth investigation. An alternative mechanism consistent with these observations is that CSE may stabilise the NOD2-RIPK2 complex, thereby preventing or retarding NFκB activation and subsequent observed reduction in chemokine production [65]. Our data suggest that CSE (or smoking) delays or prevents the prompt NOD2 upregulation in response to inflammatory stimuli and related production of chemokines that could be crucial in recruitment of inflammatory cells in the immune response to bacteria [60]. The prolonged NOD2-RIPK2 interaction with TNFα+CSE stimulation at 18 hours also suggests a delay in “turning off” the response, which might also lead to the development of chronic inflammation.

TNFα-induced production of CCL20 and IL8 at 24 hours was also reduced by CSE, but not nicotine, consistent with the patterns of NOD2 expression and NFκB activation. While this is not direct evidence of CCL20 and IL8 being affected by NOD2 expression, other studies have shown that mutations in NOD2 (and hence abnormal NOD2 expression) also led to decreased IL8 [20] and CCL20 production [52]. Similarly, pre-treatment of cells with TNFα and/or CSE prior to MDP stimulation also showed a decrease in basal and TNF-induced CCL20 and decreased basal IL8, although the effects of CSE on TNF-induced IL8 were not as strong. This suggests that CSE may differentially affect specific pathways involved in inflammation. CCL20 is involved in recruiting specific subsets of dendritic cells to the Peyer's patches in the gut [66] and is an epithelial chemokine for Th17 cells [67], [68]; both mechanisms could be important in IBD [69]. In a mouse model, cigarette smoke was found to increase apoptosis of follicle-associated epithelial cells of Peyer's Patches with increased CCL20 production [70], whereas CCL20 levels were reduced by cigarette smoke in bronchoalveolar lavage [71]and lung epithelial cells, with concomitant reduced anti-microbial activity [72]. IL8 is a chemotactic factor for neutrophils [73]; reduction of IL8 suggests that an insufficient neutrophil response may also lead to inadequate innate immunity to bacterial antigens. Neutrophil recruitment and IL8 levels in small bowel injury have been shown to be reduced in patients with CD compared with UC or healthy controls [74]. Reduction in these chemokines *in vivo* and subsequent loss of appropriate responses to e.g. AIEC would mean that these bacteria are able to cause uncontrolled inflammatory reactions [26], and thus could be further mechanisms by which smoking increases the susceptibility to small bowel CD, with its associated more aggressive disease course [42].

In HT29 but not SW480 cells, nicotine also reduced the TNFα-induced NOD2 expression. Nicotine has a variety of effects within the GI tract [35], [75] and both colonic epithelial cells and *lamina propria* T cells express nicotinic acetyl choline receptors (nAChR) [76], [77]. The differences in the cell-line responses may be due to differential expression of nAChRs, although we have previously observed nicotine modifying LPS-induced NFκB responses in SW480 cells (Aldhous, unpublished data). Animal studies of IBD have shown that nicotine exacerbates jejunal inflammation but ameliorates colonic inflammation [78], [79], suggesting that nicotine has diverse effects according to intestinal location. Given the heterogeneity of clinical presentation in IBD and the complex genetic architecture, nicotine may modulate the expression/function of susceptibility genes other than NOD2. A pilot study of nicotine enemas in CD has given limited data as regarding safety and efficacy [33] but further studies are required.

There are over 4000 compounds in cigarette smoke. As well as nicotine, we investigated three compounds each previously shown to affect NFκB activation in other experimental settings [46], [49], [50]. In the present study these chemicals did not significantly affect NFκB activity or significantly reduce TNFα-induced NOD2 expression. This does not mean they would not have effects under different experimental conditions. Individual chemicals within cigarette smoke are potentially the active compounds involved in IBD and the identification of these is desirable as a chemical target for inhibition. Alternatively, the identification of the pathway or mechanisms involved would also act as a focus for therapeutic intervention. However, the concentrations of these chemicals within smoke are hard to ascertain and the combination of constituents may be crucial in intestinal inflammation. With this in mind, we used CSE to keep the relative concentrations of components of cigarette smoke nearer to that of smoke products dissolved in saliva and swallowed [35]. There have been few studies of CSE in the gut; a study in a rat model of colitis showed that both CSE and nicotine reduced inflammation, with concomitant decrease in neutrophil activity [80]. This is consistent with our results of reduced IL8 production with CSE. CSE is widely used in research into smoking-related respiratory disease and shown to increase production of some pro-inflammatory cytokines [37], [81], but not others [47], [82] depending on the cell type and mode of stimulation. CSE has also been reported to affect apoptosis [38], neutrophil phagocytosis [39] and mechanisms of lung repair [40], [41], all of which are relevant mechanisms in the gut. Gaseous products, e.g. carbon monoxide from cigarette smoke could also have an effect. Carbon monoxide (CO) does not dissolve easily in water and may be less pertinent in CSE. CO (and biliverdin) are produced by the action of heme-oxygenase (HO-1) on heme; at low levels CO has anti-inflammatory properties [83]. In the gut, CO has been found to ameliorate colitis by the induction of IL10 via HO-1 [84] and may be the route by which aminosalicylate drugs have their effect [83].

Our aim was to investigate the inflammation-induced upregulation of NOD2 in epithelium, as these cells do not express NOD2 under homeostatic conditions [52]. The epithelial barrier increases in permeability with inflammatory cytokines [85], thus increasing access for bacteria. Decreased gut barrier function is an important feature of IBD, but whether it is due to or causative of gut inflammation [86] is not clear. Thus, upregulation of NOD2 within the cell might be important to increase antibacterial responses. Indeed, another study also showed that responses to MDP were more efficient in epithelial cells pre-treated with TNFα to induce NOD2 [52].

In conclusion, we report that CSE reduced TNFα-induced NOD2 expression, possibly by inhibiting upstream NFκB translocation, that CSE affected NOD2 signalling and reduced chemokine production. These data provide novel evidence for potentially important mechanisms whereby smoke may affect intestinal inflammation and modulate the phenotype of CD. Indeed the implications and relevance of these findings may extend widely beyond IBD as the importance of NOD2 in other diseases processes is better understood.
